# Integrated DNA methylation analysis reveals a potential role for *ANKRD30B* in Williams syndrome

**DOI:** 10.1038/s41386-020-0675-2

**Published:** 2020-04-18

**Authors:** Ryo Kimura, Roy Lardenoije, Kiyotaka Tomiwa, Yasuko Funabiki, Masatoshi Nakata, Shiho Suzuki, Tomonari Awaya, Takeo Kato, Shin Okazaki, Toshiya Murai, Toshio Heike, Bart P. F. Rutten, Masatoshi Hagiwara

**Affiliations:** 1 Department of Anatomy and Developmental Biology, Graduate School of Medicine, Kyoto University, Kyoto, Japan; 2 Department of Psychiatry and Psychotherapy, University Medical Center Göttingen, Göttingen, Germany; 3 Department of Psychiatry, McLean Hospital, Harvard Medical School, Belmont, MA USA; 4 Department of Pediatrics, Graduate School of Medicine, Kyoto University, Kyoto, Japan; 5 Department of Child Neurology, Osaka City General Hospital, Osaka, Japan; 6 Todaiji Ryoiku Hospital for Children, Nara, Japan; 7 Department of Cognitive and Behavioral Science, Graduate School of Human and Environmental Studies, Kyoto University, Kyoto, Japan; 8 Department of Psychiatry, Graduate School of Medicine, Kyoto University, Kyoto, Japan; 9 Department of Pediatrics, Hyogo Prefectural Amagasaki General Medical Center, Amagasaki, Japan; 10 Division of Neuroscience, School for Mental Health and Neuroscience (MHeNS), Department of Psychiatry and Neuropsychology, Maastricht University Medical Centre, Maastricht, The Netherlands

**Keywords:** DNA methylation, Autism spectrum disorders, Translational research

## Abstract

Williams syndrome (WS) is a rare genetic disorder, caused by a microdeletion at the 7q11.23 region. WS exhibits a wide spectrum of features including hypersociability, which contrasts with social deficits typically associated with autism spectrum disorders. The phenotypic variability in WS likely involves epigenetic modifications; however, the nature of these events remains unclear. To better understand the role of epigenetics in WS phenotypes, we integrated DNA methylation and gene expression profiles in blood from patients with WS and controls. From these studies, 380 differentially methylated positions (DMPs), located throughout the genome, were identified. Systems-level analysis revealed multiple co-methylation modules linked to intermediate phenotypes of WS, with the top-scoring module related to neurogenesis and development of the central nervous system. Notably, *ANKRD30B*, a promising hub gene, was significantly hypermethylated in blood and downregulated in brain tissue from individuals with WS. Most CpG sites of *ANKRD30B* in blood were significantly correlated with brain regions. Furthermore, analyses of gene regulatory networks (GRNs) yielded master regulator transcription factors associated with WS. Taken together, this systems-level approach highlights the role of epigenetics in WS, and provides a possible explanation for the complex phenotypes observed in patients with WS.

## Introduction

Williams syndrome (WS, OMIM 194050), also referred to as Williams–Beuren syndrome, is a rare genetic disorder caused by the heterozygous deletion of 26–28 genes at 7q11.23, occurring in ~1 of 10,000 individuals [1]. The disorder is well known for distinctive symptoms encompassing physical and cognitive features and abilities, including characteristic facial features, cardiovascular abnormalities, endocrine imbalances, and intellectual disability [1]. In particular, hypersociability and over-friendliness to strangers, so called “cocktail party” personality, have received considerable attention [1, 2]. Such hypersocial behavior seems to be opposite to autism spectrum disorders (ASD), which are characterized by reduced interest in social stimuli and genetic heterogeneity [2]. Therefore, studying WS is expected to illuminate genes and pathways relevant to social behavior [2]. Several attempts to identify the functions of deleted genes have suggested that *GTF2I* is implicated in behavioral and/or cognitive abnormalities [3]. However, this does not explain the phenotypic variability observed among WS patients, and genes related to neuropsychiatric features such as hypersociability remains largely unknown.

Epigenetic mechanisms such as DNA methylation or histone modification have been implicated in an array of neuropsychiatric disorders, and thus might mediate social behavior [4, 5]. In particular, DNA methylation significantly modifies transcriptional regulation by altering transcription factor binding sites and chromatin accessibility [6]. Accumulating evidence indicates that altered DNA methylation in the blood, for example, in the gene encoding the oxytocin receptor, is associated with social behavior, implying that epigenetic effects on social behavior are detectable even in peripheral blood [7]. Intriguingly, in WS, several genes that are deleted from 7q11.23 are linked to epigenetic regulation [8]. For example, *BAZ1B*, *BCL7B*, and *WBSCR22* are implicated in chromatin remodeling [9–11]. However, the potential role of DNA methylation in WS is poorly characterized. To date, only one pioneering study has reported a potential role of DNA methylation in WS, though that cohort was limited [8]. Therefore, larger studies based on multiple experimental approaches are needed to elucidate the role of DNA methylation in WS.

We used a relatively large sample of WS patients and multiple methods to address this need. We used robust methylome-wide measurement methods, and employed weighted gene co-methylation network analysis (WGCNA), an unbiased, systems biology approach widely used to elucidate underlying biological mechanisms [12]. Furthermore, we integrated methylation and gene expression profiles using samples from the same individuals to construct gene regulatory networks (GRNs) and identify master regulator transcription factors related to WS [13]. Together, our findings provide novel biological insights into the role of epigenetics in WS.

## Materials and methods

### Samples and assessments

Ninety WS patients were recruited from each pediatric neurology department (Kyoto University Hospital, Osaka City General Hospital, and Todaiji Ryoiku Hospital for Children) through the WS family support group in Japan. Diagnosis of WS was established by fluorescent in situ hybridization (FISH). Thirty-four healthy individuals as controls were recruited from the general community through advertising. Controls were healthy people without a current or previous diagnosis of a psychiatric disorder, and had a negative family history for WS. Functional behavior was assessed according to Japanese versions of the Child Behavior Checklist for ages 6–18 (CBCL) or the Adult Behavior Checklist (ABCL) [14]. Social functions were evaluated using the Social Responsiveness Scale-2 (SRS-2); total *T*-scores of 59 or less indicated no clinically significant concerns in social functioning, whereas 60–65 indicated mild to moderate deficits in social interaction [15]. *T*-scores were determined for both behavior and social function, and the questionnaires for patients were completed by their parents. All participants were Japanese, with no history of use of psychotropic medications, did not smoke, and had not received any medication for at least three months before the collection of the blood samples. Whole-blood samples were collected in EDTA tubes. Genomic DNA was extracted with the QIAamp DNA Blood Midi Kit (Qiagen, Tokyo, Japan) and treated with RNase A (Qiagen) to remove potential RNA contamination. DNA quality was assessed using a NanoDrop 2000 spectrophotometer (Thermo Fisher Scientific, Yokohama, Japan) and with an Agilent 2200 TapeStation (Agilent Technologies, Tokyo, Japan). All blood samples were collected on the same day as clinical assessments. All patients were confirmed to have typical 7q11.23 deletions based on genomic quantitative PCR as previously described [16]. This study was approved by institutional ethics committees at each participating institution. Written informed consent was obtained from all participants and/or their parents according to the Declaration of Helsinki. Detailed demographics are provided in Supplementary Table S1.

### Genome-wide DNA methylation profiling

Genome-wide DNA methylation profiling was conducted on blood samples from 34 WS patients and 34 controls. Genomic DNA was bisulfite-converted using the EZ-96 DNA Methylation Kit (Zymo Research, Irvine, CA, USA). DNA methylation was assessed using Illumina Infinium HumanMethylation450 BeadChip (Illumina, San Diego, CA, USA). Data quality control and analysis were performed with ChAMP version 2.6.4 [17]. Briefly, probe filtering was applied to remove probes that failed to hybridize with a detection *p*-value (*p*) above 0.01 in one or more samples and with a bead count of <3 in 5% of samples; then, probes on sex chromosomes, those containing single-nucleotide polymorphisms, and non-specific binding probes were also filtered out [17]. The *β*-value distributions of the two types of probes were corrected using Beta Mixture Quantile dilation [18]. After quality control, 408,525 CpG probes were retained for further analysis. The singular value decomposition (SVD) method was used to identify batch effects and the ComBat method was used to correct for their effects related to age, sex, array slide, and position. Cell-type composition was estimated using the Houseman algorithm to adjust for potential differential cellular heterogeneity in blood [19]. Differentially methylated positions (DMPs) with a false discovery rate (FDR) < 0.05 and *β*-value difference >0.1 were identified using the R package Limma [20]. Differentially methylated regions (DMRs) were identified using the DMRcate R package with the default settings [21]. DMRcate is based on tunable kernel smoothing of the differential methylation signal. DMRs were defined as regions with a maximal 1000 bp containing two or more CpGs. Multiple testing correction was performed by the Benjamini–Hochberg procedure for FDR [22]. The gometh function of the R package missMethyl was used to analyze gene ontology (GO) enrichment in the original list of 408,525 CpG sites [23].

### Weighted gene co-methylation network analysis (WGCNA)

The WGCNA R package was applied to the entire methylation dataset to identify co-methylation modules [12]. Briefly, to construct modules, the soft-thresholding power was set to 12 to maximize scale-free topology model fit as it plateaued above 0.9. The initial module assignments were determined by using a dynamic tree-cutting algorithm with deepSplit = 4, minModulesize = 100, and dthresh = 0.2. The methylation profile of each module was summarized using the first principal component (the module eigengene). Module eigengenes were then analyzed for correlation with sample traits. Genes with the highest module membership values were considered hub genes. Modules were characterized by GO using Enrichr [24]. Details of disease enrichment analyses are provided in the Supplemental Data.

### Pyrosequencing

Pyrosequencing was performed on the samples from the methylation set (34 WS patients and 34 controls) as well as the complete WS set (90 WS patients). DNA was bisulfite-converted using the EpiTect Fast DNA Bisulfite Kit (Qiagen) and amplified by PCR using the PyroMark PCR kit (Qiagen). Primers were designed using PyroMark Assay Design Software 2.0 (Qiagen) and are listed in Supplementary Table S2. PCR conditions consisted of initial denaturing at 95 °C for 15 min; 45 cycles of 94 °C for 30 s, 56 °C for 30 s, and 72 °C for 30 s; and final extension at 72 °C for 10 min. Pyrosequencing was performed on the PyroMark Q96 ID system with Gold Q96 Reagents (Qiagen). Methylation at each CpG site was analyzed with PyroMark Q96 software (Qiagen). Mann–Whitney *U* test was used for significance test.

### Correlation analysis

DNA methylation was compared between blood samples and brain tissues using the Blood Brain DNA Methylation Comparison Tool (https://epigenetics.essex.ac.uk/bloodbrain/ [25]). Correlations between DNA methylation and age were assessed based on the Spearman’s correlation coefficient.

### Enhancer linking by methylation/expression relationships (ELMER) analysis

Using the ELMER R package (version 2.6.1) in supervised mode, the methylation and expression data were integrated to investigate GRNs and identify related master regulator transcription factors [13]. Briefly, for this analysis the processed methylation data *β*-values, as described above, were used, combined with published expression data [16]. More details are provided in the Supplemental Data.

### Availability of data and materials

DNA methylation data are available at NCBI Gene Expression Omnibus (https://www.ncbi.nlm.-nih.gov/geo/) under accession number GSE119778. The code used to analyze the data is available from the corresponding author upon reasonable request.

## Results

### Genome-wide DNA methylation profiles

**Fig. 1:**
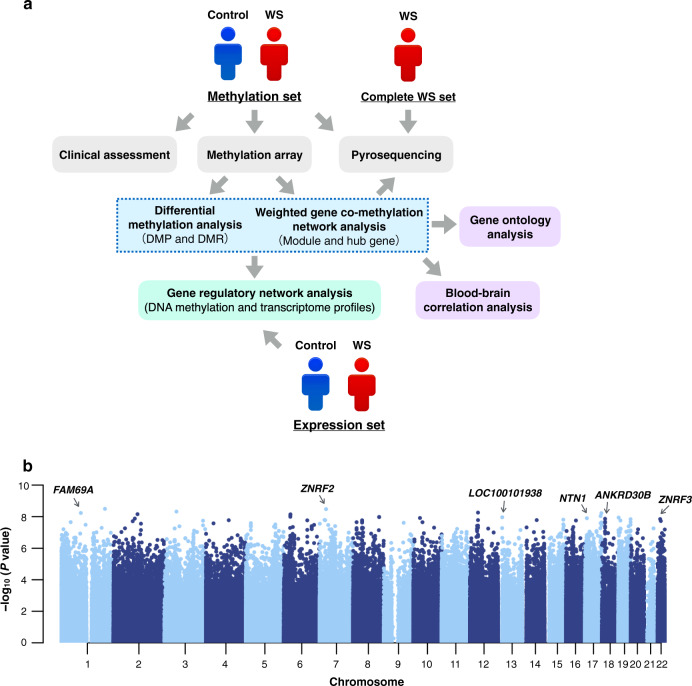
Genome-wide DNA methylation profiles of WS patients and control individuals. **a** Overview of the study. **b** Manhattan plot showing chromosomal locations of −log10 (*p*-values) for the association at each locus. Top DMPs in the methylation set are annotated by gene symbols.

An overview of the study design is shown in Fig. 1a. The methylation set was balanced to ensure no significant differences in age and sex between WS patients and controls. Total *T*-scores on the SRS-2 were significantly higher in WS patients than in controls, and profiles based on the CBCL/ABCL were also significantly more severe, whereas changes in the social motivation subscale were not significant (Supplementary Table S1).

We investigated DNA methylation differences between WS patients and controls using methylation arrays. After adjusting for possible confounding factors, multidimensional scaling plots clearly separated WS patients and controls (Supplementary Fig. S1). SVD revealed that the first principal component (PC) was significantly associated with the study groups (Supplementary Fig. S1). In addition, white blood cell proportions were estimated using the Houseman method [19]. We found that the number of B cells, CD4^+^ T cells, and granulocytes was significantly different between patients and controls (Supplementary Fig. S2). We then adjusted for the estimated cell-type proportions using linear regression models. We identified 380 DMPs with an FDR < 0.05 and *β*-value difference >0.1 (Supplementary Table S3), with top-scoring sites located in or near *ZNRF2*, *FAM69A*, *LOC100101938*, *NTN1*, *ANKRD30B*, and *ZNRF3* (Table 1A). The corresponding Manhattan plot showed that these positions were distributed across the genome (Fig. 1b). Global methylation status of DMPs indicated that WS patients exhibited significantly higher methylation levels than controls (Supplementary Fig. S3). DNA methylation differences at each position within the deleted region were small and below 0.1 (Supplementary Table S4). GO analysis of biological process (Supplementary Fig. S4 and Supplementary Table S5) suggested that hypermethylated sites were significantly enriched in homophilic cell adhesion (*p* = 4.05E−8) and cell–cell adhesion via plasma membrane (*p* = 1.93E−6). In contrast, hypomethylated sites were enriched for protein localization to membrane (*p* = 2.16E−4) and negative regulation of adenylate cyclase (*p* = 2.16E−4).

**Table 1:** (A) A list of genome-wide significant differentially methylated positions (DMPs). (B) List of genome-wide significant differentially methylated regions (DMRs).

(A) CpG site	Gene symbol	Chr.	Feature	*β* in WS	*β* in control	Δ*β*	*p-*value
cg24152511	NA	chr1	IGR	0.28	0.40	−0.12	3.19E−09
cg08549335	*ZNRF2*	chr7	Body	0.20	0.35	−0.15	3.34E−09
cg11310341	NA	chr12	IGR	0.55	0.37	0.18	5.54E−09
cg08893087	*FAM69A*	chr1	Body	0.57	0.69	−0.12	5.85E−09
cg15915602	NA	chr2	IGR	0.83	0.68	0.15	6.98E−09
cg16519217	NA	chr6	IGR	0.52	0.64	−0.12	8.11E−09
cg15509177	*LOC100101938*	chr13	TSS200	0.72	0.55	0.17	1.10E−08
cg01862363	*NTN1*	chr17	Body	0.67	0.53	0.14	1.26E−08
cg21293934	*ANKRD30B*	chr18	TSS200	0.65	0.45	0.20	1.37E−08
cg07127410	*ZNRF3*	chr22	Body	0.50	0.61	−0.11	1.43E−08

(B) Chr.	Start (bp)	End (bp)	CpG	FDR	Stouffer	Mean *β*	Overlappingpromoters
chr7	27141388	27144595	30	1.47E−44	4.90E−25	−0.02	*HOXA2*
chr6	49681178	49681774	9	1.97E−39	4.18E−20	0.09	*CRISP2*
chr6	3848218	3850738	34	1.11E−29	8.18E−19	0.02	*FAM50B*
chr6	28058715	28059208	9	2.23E−36	1.13E−18	0.06	*ZSCAN12P1*
chr18	14747036	14748439	14	1.80E−62	2.28E−18	0.11	*ANKRD30B*

*IGR* intergenic region, *Body* gene body, *TSS200* 0–200 bases upstream from the transcriptional start site, *FDR* false discovery rate, *Stouffer* Stouffer’s *p*-value, *NA* not available.

Apart from individual CpG sites, we next sought to identify DMRs spanning multiple CpG probes using DMRcate. *HOXA2* and *CRISP2* were identified, for which the Stouffer’s *p*-values were 4.90E−25 and 4.18E−20, respectively (Table 1B, Supplementary Table S6). Of particular interest, *ANKRD30B* was one of the most significant DMRs, with a Stouffer’s *p*-value of 2.28E−18 (Table 1B) and containing top-scoring DMPs (Table 1A).

### Identification of co-methylation modules related to WS

**Fig. 2:**
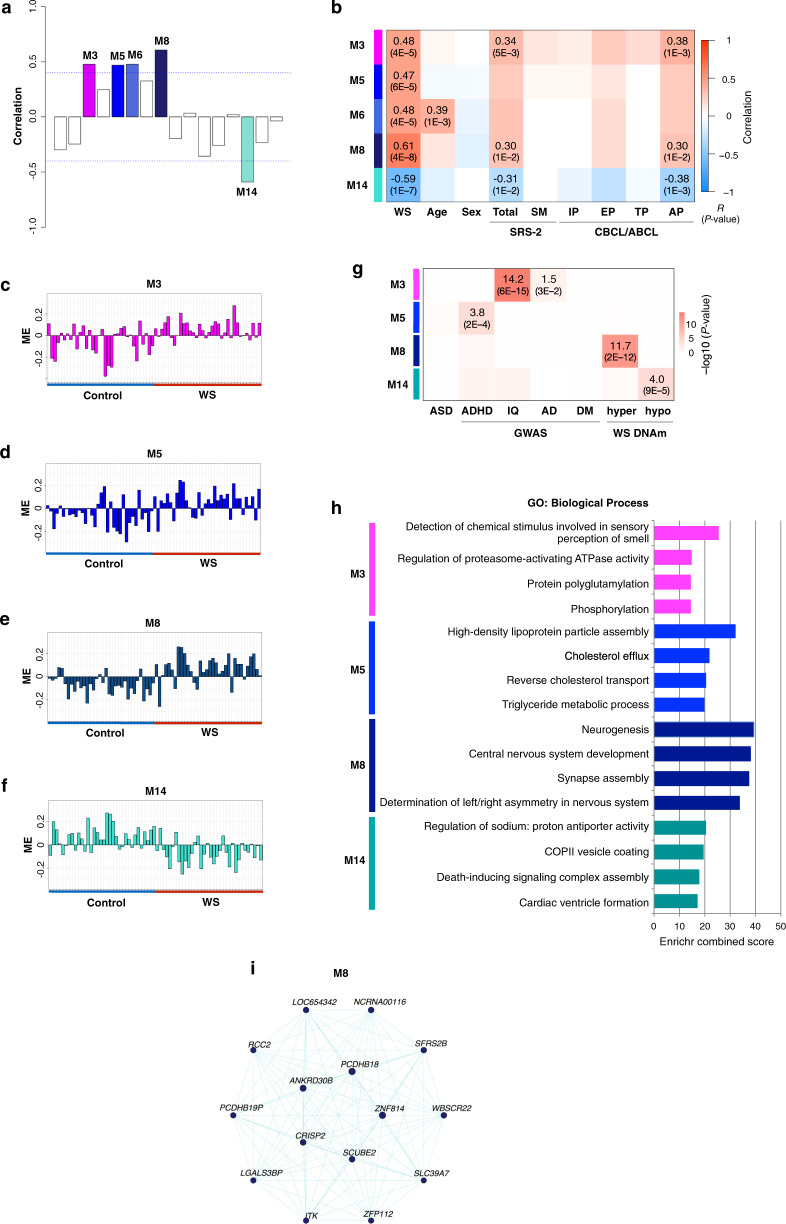
Co-methylation modules associated with WS. **a** Correlation between module eigengenes and WS. Colored bars represent significant correlations with an FDR < 0.05 and an absolute *R* > 0.45. **b** Heatmap of positive (red) or negative (blue) correlations between modules and traits. SRS-2 Social Responsiveness Scale Second Edition, Total total *T*-score, SM social motivation, CBCL/ABCL Child/Adult Behavior Checklist, IP internalizing problems, EP externalizing problems, TP thought problems, AP attention problems. **c**–**f** Module eigengene values (*y* axis) for **c** M3, **d** M5, **e** M8, and **f** M14 in individual samples (*x* axis). **g** Enrichment analysis of disease-associated genes. Gene lists were compiled from the literature (Supplementary Table S7). ASD autism spectrum disorder, ADHD attention-deficit hyperactivity disorder, IQ intelligence quotient, AD Alzheimer disease, DM diabetes mellitus, hyper and hypo hypermethylated and hypomethylated genes in WS. **h** Top four biological processes associated with each module. GO terms are ranked based on the Enrichr combined score. **i** Co-methylation networks among hub genes in M8. The FDR < 0.05 are shown in parentheses.

To acquire a systems-level understanding of the relationship between DNA methylation and WS, we analyzed correlation patterns among *β*-values across the entire dataset by WGCNA. We identified 16 co-methylation modules that could be described by module eigengenes (Supplementary Fig. S5). Five of these were found to be significantly correlated with WS with an FDR < 0.05 and absolute *R* > 0.45 (Fig. 2a, b). Of these, module M8 was the most strongly correlated (*R* = 0.61 and FDR = 4.19E−8; Fig. 2b). Notably, three of these modules (M3, M8, and M14) were significantly correlated with total *T*-scores on the SRS-2 and attention problems on the CBCL/ABCL (Fig. 2b). In contrast, M6 was significantly correlated with age, a known confounding factor, and hence was excluded from subsequent analysis. M3, M5, and M8 contained genes that were hypermethylated in WS patients, whereas those of M14 were hypomethylated (Fig. 2c–f). Together, we successfully identified multiple WS-associated modules, which may potentially link to their phenotypes.

To explore the possible link between WS-associated modules and genetic findings in neuropsychiatric disorders, we performed gene list enrichment analysis (Supplementary Table S7). The M3 module was significantly enriched for intelligence- and Alzheimer disease-associated genes (FDR = 2.9E−2 and 6.2E−15, respectively; Fig. 2g). The M5 module was significantly enriched for ADHD (FDR = 1.6E−4), whereas M8 and M14 were significantly enriched in differentially methylated genes previously identified in WS (FDR = 2.1E−12 and 9.0E−5, respectively). To further investigate the biological function of each module, GO was analyzed using Enrichr (Fig. 2h, Supplementary Table S8). Remarkably, M8 was highly enriched in genes associated with neurogenesis, central nervous system development, and synapse assembly. The top 15 connected genes in this module as hubs included *ANKRD30B*, *WBSCR22*, and *PCDHB18* (Fig. 2i, Supplementary Table S9). M3 was enriched for genes related to the detection of chemical stimuli involved in the sensory perception of smell, with *SNRNP70* and *TRIM40* as top hub genes (Supplementary Fig. S5). M5 was enriched in genes related to lipoprotein and cholesterol, with *PDE9A* and *FBN3* as hub genes (Supplementary Fig. S5). M14 was enriched in genes that regulate sodium levels and cardiac ventricle formation, with *RGS2* and *DUSP26* as hub genes (Supplementary Fig. S5). Taken together, these findings suggest that M8 might be disease-specific, thereby providing potential insight into the complex neuropsychiatric phenotypes of WS.

### *ANKRD30B* methylation and expression in WS

**Fig. 3:**
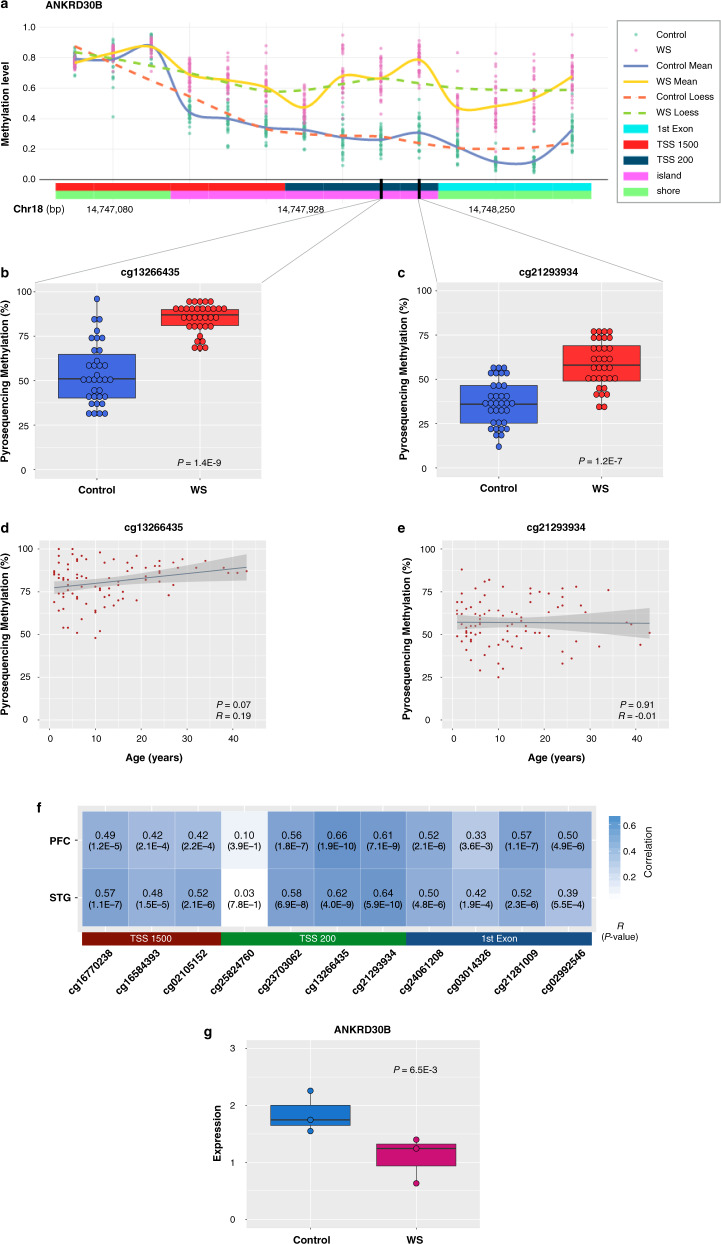
Targeted pyrosequencing of *ANKRD30B* and correlation between *ANKRD30B* methylation in the blood and brain. **a** Scatter plots with loess lines showing *ANKRD30B* methylation in WS patients and control individuals. Data points are methylation levels for each probe in individual samples based on methylation array. **b**, **c** Validation of selected CpG sites in *ANKRD30B*. Box plots represent methylation levels as measured by pyrosequencing. Groups in the methylation set (34 WS patients and 34 controls) were compared by Mann–Whitney *U* test. **d**, **e** Effects of age on DNA methylation in *ANKRD30B* using the complete WS set (90 WS patients). Spearman’s correlation coefficient (*R*) and *p*-values are indicated. **f** Correlation of the methylation status of CpG sites in *ANKRD30B* between blood samples and PFC and STG tissues in the brain. Pearson’s correlation coefficient (*R*) and *p*-values are indicated. TSS1500 200–1500 bases upstream from the transcriptional start site, TSS200 0–200 bases upstream from the transcriptional start site. **g** Differential expression of *ANKRD30B*. *ANKRD30B* was significantly downregulated in WS patients compared to controls in frontal cortex. RNA-Seq data (GSE128841) were obtained from Barak et al. [3]. DESeq was used for differential expression analysis. Pink dots indicate WS patients (*n* = 3), whereas blue dots indicate controls (*n* = 3).

We then investigated *ANKRD30B* in M8, as it was identified in both conventional and systems-level approaches (Figs. 2i and 3a). Accordingly, *ANKRD30B* was pyrosequenced in the methylation set using probes against transcription start sites, as such sites are more likely to affect the expression of downstream genes or phenotypes. We found that cg13266435 and cg21293934, both of which are located in the *ANKRD30B* promoter, were hypermethylated in WS patients (*p* < 1.4E−9 and *p* < 1.2E−7, respectively; Fig. 3b, c). Other CpG sites were also significantly hypermethylated (Supplementary Fig. S6). As it is known to have a clear effect on DNA methylation, we assessed whether age would influence DNA methylation of *ANKRD30B* in WS. Using the complete WS set consisting of 90 patients with an age range of 1–43 years old, we found that age was not significantly correlated with *ANKRD30B* methylation levels at both at either cg13266435 or cg21293934 (Fig. 3d, e). In addition, we examined the effects of sex on DNA methylation in *ANKRD30B* within WS patients. Although *ANKRD30B* methylation levels at cg13266435 were not significantly different between male and female patients, methylation levels at cg21293934 were weakly significantly different (*p* = 0.54 and *p* = 0.04, respectively; Supplementary Fig. S7).

Next, the methylation state of each CpG site in *ANKRD30B* was compared between blood and brain tissues using the Blood Brain DNA Methylation Comparison Tool [25]. Ten out of eleven CpG sites identified in the blood samples were significantly and positively correlated with both the prefrontal cortex (PFC) and the superior temporal gyrus (STG) regions (*R* = 0.33–0.66 and *R* = 0.39–0.64, respectively; Fig. 3f). It is known that both PFC and STG regions are related to social function. Recently, multiple studies have shown that DNA methylation at specific loci can be influenced by sequence variations, and such sites are called methylation quantitative trait loci (meQTL) [26]. However, we found that *ANKRD30B* sites were not associated with meQTL in the developing brain (Supplementary Table S10) [25].

Furthermore, we examined expression of *ANKRD30B* in frontal cortex brain tissues from WS patients (*n* = 3) and controls (*n* = 3; GSE128841) [3]. Although the sample size was small, we found that *ANKRD30B* was significantly downregulated in WS patients compared to controls (Fig. 3g). Together, these results suggest that *ANKRD30B* hypermethylation might occur not only in the blood but also in the brain.

### Integrative analysis of gene expression and methylation

**Fig. 4:**
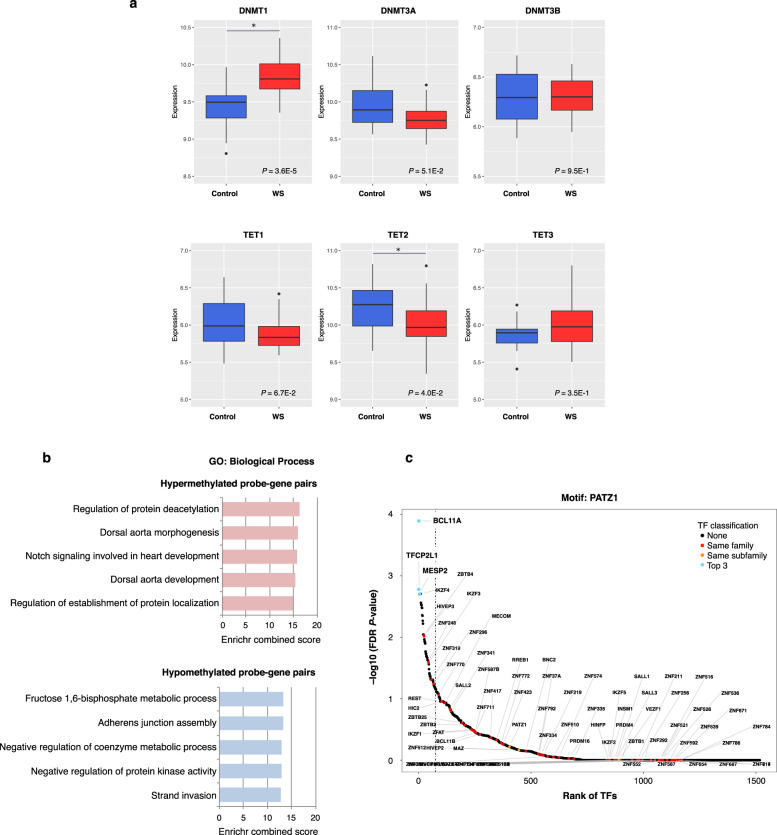
Gene expression profiles of DNA methylation regulators and gene regulatory networks related to WS. **a** Epigenetic regulators (*DNMT1*, *DNMT3A*, *DNMT3B*, *TET1*, *TET2*, and *TET3*) expression levels were determined by our published data (GSE89594) [16]. Red indicates WS patients (*n* = 32), whereas blue indicates controls (*n* = 30). The FDR < 0.05 was considered significant. **b** Top biological process terms associated with hyper- and hypomethylated probe–gene pairs. GO terms are ranked based on the Enrichr combined score. **c** Identifi**c**ation of master regulator transcription factors related to WS. All transcription factors are ranked by their correlation with methylation changes of distal probes of a *PATZ1*-binding motif, which *BCL11A* binds. *Colored dots* indicate the top three most anti-correlated transcription factors (*BCL11A*, *TFCP2L1*, and *MESP2*). The *vertical dotted line* indicates the 5% highest ranked transcription factors. FDR false discovery rate, TF transcription factor.

We next investigated possible changes in the DNA methylation/demethylation machinery in WS. DNA methylation patterns are established and maintained by DNA methyltransferases (DNMTs) such as DNMT1, DNMT3A, and DNMT3B, whereas the ten-eleven translocation (TET) family including TET1, TET2, and TET3 mediates active DNA demethylation [6]. Epigenetic regulators (*DNMT1*, *DNMT3A*, *DNMT3B*, *TET1*, *TET2*, and *TET3*) expression levels were determined using our previously published gene expression data (GSE89594) [16]. Interestingly, we found that *DNMT1* was significantly upregulated, whereas *TET2* was significantly downregulated in WS patients (Fig. 4a). These findings may provide clues to further our understanding of the mechanisms behind aberrant methylation in WS.

Finally, to investigate GRNs and related master regulator transcription factors, we performed ELMER analysis with 141,731 distal probes. After the formation of potential probe–gene pairs, 18 of the hypermethylated probe–gene pairs and 62 of the hypomethylated probe–gene pairs were found to be significantly associated (Supplementary Table S11). The top GO biological processes associated with the genes in these pairs are shown in Fig. 4b; Supplementary Table S12. Notably, the hypermethylated probe–gene pairs were enriched for dorsal aorta morphogenesis and heart development. In contrast, the hypomethylated probe–gene pairs were enriched in fructose metabolic process and adherens junction. Subsequent motif enrichment analysis identified no motifs associated with the hypermethylated probes, whereas 44 motifs were enriched with the hypomethylated probes (Supplementary Table S13), thus only the latter were used for the identification of upstream transcription factors. The top three transcription factors associated with the hypomethylated probes in WS were *BCL11A* (*p* = 1.29E−04), *TFCP2L1* (*p* = 1.68E−03), and *MESP2* (*p* = 1.97E−03; Fig. 4c).

## Discussion

The primary aim of this study was to identify DNA methylation profiles that are associated with WS using peripheral blood. Among several co-methylated modules identified, *ANKRD30B* (ankyrin repeat domain-containing protein 30B) was found to be significantly hypermethylated in patients, both by conventional and systems-level analysis. Importantly, we found that *ANKRD30B* methylation status in blood samples was not only similar to that in brain tissues, but also elevated over a wide age range. Furthermore, we identified master regulator transcription factors related to WS by integrating methylation and gene expression profiles.

*ANKRD30B* is located at 18p11.21, and is known to be more abundantly expressed in the brain than in blood [27]. In our study, we could not detect any expression of the *ANKRD30B* gene in the blood samples from both WS patients and controls. Although its biological function is unclear, a recent study showed that it is hypermethylated and downregulated in the brains of Alzheimer disease patients [28]. This result might provide a epigenetic insight into the observation that some WS patients exhibit early cognitive decline like senile dementia [1]. Interestingly, sequence similarity analysis of *ANKRD30B* among species revealed that only *ANKRD30B* of gorilla and vervet-AGM are highly conserved with humans throughout evolution with high confidence (Supplementary Table S14). Thus, *ANKRD30B* might have a critical role in unique brain functions in higher primates, although this requires experimental validation. Other top DMRs, such as *HOXA2* and *CRISP2*, also showed an association with disease phenotypes. The transcriptional regulator *HOXA2* (Homeobox A2) controls the development of some parts of the face and middle ear [29], whereas *CRISP2* (Cysteine-rich secretory protein 2), also identified as a hub gene in M8, is a testicular sperm protein proposed to be involved in sperm-egg interaction [30]. *CRISP2* is differentially methylated in atherosclerotic patients, suggesting an association with cardiovascular diseases [31]. Although we found that nine CpG sites in *CRISP2* identified in blood were significantly and positively correlated with both the PFC and STG regions (Supplementary Fig. S8), the expression levels of *CRISP2* in the brain from WS patients remain unknown. As WS patients present characteristic facial features and hearing problems such as hyperacusis, and are commonly hypertensive [1], these data collectively highlight aberrant DNA methylation as a potential underlying cause.

Unlike conventional methods, WGCNA mines gene networks constructed by the hierarchical clustering of methylated sites [12]. Four modules identified in this manner (M3, M5, M8, and M14) were found to be associated with WS. Although blood samples were tested, GO analysis indicated that M8 encompasses genes associated with neuronal development. In addition, M3 was associated with olfactory sensation, intelligence, and Alzheimer disease. Although olfactory impairment has been linked to prodromal Alzheimer disease [32], olfactory functions in WS are still poorly characterized compared to visual and hearing issues. Meanwhile, M5 was associated with metabolic processes such as lipoprotein and cholesterol, which might provide clues as to why adults with this disease tend to develop obesity [1]. Therefore, our identified co-methylation modules have the potential to contribute to understanding the epigenetic basis of WS phenotypes.

Cardiovascular abnormalities like supra valvular aortic stenosis seen in WS are considered to be caused by the deletion of elastin [1]. However, the involvement of epigenetic factors has been suspected because not all WS patients present with cardiac abnormalities. Our results showed that the hypermethylated probe–gene pairs were associated with dorsal aorta morphogenesis and heart development, further supporting the idea that epigenetic events contribute to the cardiovascular abnormalities seen in WS.

Furthermore, our approach revealed that an unexpected phenotype that could be related to WS. Recently, Burkitt lymphoma, a highly aggressive B-cell non-Hodgkin type, has been gaining interest in terms of its relationship with WS [33]. We found that B cells were significantly more prevalent in patients than in controls. This result supports the concept that abnormal B cell counts in WS patients can result in lymphoma. However, it should be noted that cell-type compositions in this study were estimated by the Houseman algorithm. Therefore, further study will be needed to confirm with hematologic assessments using cell staining and to search for unidentified mutations in WS.

DNA methylation is influenced by genetic, environmental and technical factors such as ethnicity, age, smoking status, and medication. Thus, the ability to replicate findings of past methylation studies might be limited, even if some of these factors are controlled. The present study primarily analyzed Japanese adolescent and young adult individuals, whereas the previous study examined children approximately 5–6 years of age and mainly of European ancestry [8]. Despite these differences, the top DMPs at or near *ANKRD30B, NTN1*, *RFPL2*, and *PRDM9* overlap those identified previously in children with WS [8]. *NTN1* is implicated in axonal outgrowth and guidance, and thus is involved in the development of the nervous system [34]. *RFPL2* is abundantly expressed in the developing human neocortex and during the onset of neurogenesis [35], whereas *PRDM9* positions double-strand breaks during homologous recombination [36]. Our findings extend those of the previous study and provide robust evidence that alterations in DNA methylation patterns occur in WS.

Gene expression levels are strongly influenced by the methylation levels at distal regulatory elements, notably enhancers [37]. The ELMER analysis exploits the interplay between DNA methylation and transcription factor binding at distal regulatory sites to identify potential master regulator transcription factors that may help explain how microdeletion has such widespread functional consequences. Therefore, identifying master regulator transcription factors provides insight into complex biological outcomes through the regulation of multiple genes, and these transcription factors could serve as targets for the development of novel therapeutic approaches [38]. Through ELMER analysis, we identified *BCL11A* as a potential upstream master regulator transcription factor. Interestingly, this gene encodes subunits of SWI/SNF chromatin-remodeling complexes, known to be involved in intellectual disability and previously identified as hub genes in an upregulated module in WS [16]. A causal link between aberrant methylation throughout the genome and deletion of 7q11.23 remains to be elucidated. Several genes within the 7q11.23 region that have been shown to be associated with chromatin remodeling may provide clues [9–11]. Notably, *WBSCR22* (also known as *BUD23*), containing a methyltransferase domain, is thought to act on DNA methylation [10]. Therefore, it is possible that the loss of these genes could trigger in an epigenetic imbalance in WS, especially as histone modifications interact with DNA methylation to modify chromatin remodeling.

Social behavior is essential for human and animal well-being, and is mediated by multiple brain regions such as the medial PFC, amygdala, and superior temporal sulcus [2]. Of these, the first is generally thought to be related to social information processing and integration, whereas the last mediates social cognition including the perception of faces and human emotion [39, 40]. Interestingly, brain imaging has shown that abnormal PFC activity, including that in the orbitofrontal cortex, is linked to hypersociability in WS patients [41]. Further challenges of WS remain to reveal the relationships between DNA methylation and brain activity on imaging.

Our study has several limitations. As WS is rare, high-quality age- and sex-matched brain and other tissues are difficult to collect, and thus, surveys are based solely on peripheral blood. Here we analyzed blood samples from age- and sex-balanced Japanese subjects. However, a potential bias should be considered, as adolescent individuals were included in the WS patient group but do not in the controls, and the effects of genetic background/ancestry were not examined. Further, other important factors might be undetectable in our survey due to the limited coverage of CpG sites in the genome on the Illumina 450K array. Accordingly, methyl-seq might prove informative in the future, especially if costs are further reduced. Last, we used the SRS-2 based on a parental/caregiver questionnaire, which is widely used as an efficient quantitative measure of the various dimensions of social behavior [15]. Our results demonstrated that WS patients have mild–moderate impairments in social functioning, according to this metric. These results were consistent with a previous clinical study [42]. However, as patients have intellectual disability, they could not complete the tests by themselves. Therefore, we had no choice but to evaluate their behavior from mixed datasets, by self-assessment (for control subjects) and by parental-assessment (for patients).

To our knowledge, this is the largest survey of DNA methylation in WS. Altered methylation patterns in blood samples were observed to converge on several disease-specific modules. Although the present study provides novel evidence regarding epigenetic imbalances based on DNA methylation and gene dysregulation in WS, further mechanistic studies are required to fully understand how these epigenetic alterations lead to complex behavioral phenotypes.

## Funding and disclosure

The authors declare no competing interests.

## Supplementary information

Supplementary Information

APC Form
