# Supplementary material for: Integrated DNA methylation analysis reveals a potential role for ANKRD30B in Williams syndrome
Source: Neuropsychopharmacology. 2020 Apr 18;45(10):1627–36. doi: 10.1038/s41386-020-0675-2 (PMC7419304; doi:10.1038/s41386-020-0675-2)
Supplement: Supplementary file 1 — Supplementary Information [file 41386_2020_675_MOESM1_ESM.docx]

**Integrated DNA methylation analysis reveals a potential role for *ANKRD30B* in Williams syndrome**

**Supplementary Information**

**This file includes:**

**1. Supplementary Methods**

**2. Supplementary Figures 1-8**

**3. Supplementary References**

**4. Supplementary Data Files – available in separate Excel sheets**

**1. Supplementary Methods**

***1.1 Enrichment analysis of disease-associated genes***

We examined the enrichment of genes associated with neuropsychiatric disorders in each module as previously described [1]. Gene lists were obtained from literature sources as detailed in Table S7. ASD genes were curated as highly validated genes by Parras et al. [2]. ADHD candidate genes were obtained from Demontis et al. [3]. Intelligence quotient candidate genes were obtained from Savage et al. [4]. Alzheimer disease candidate genes and Diabetes Mellitus candidate genes were obtained from Nardone et al. [5]. Hypermethylated and hypomethylated genes with DMRs in WS were obtained from Strong et al. [6]. A two-sided Fisher’s exact test was used to assess the significance.

***1.2 Enhancer linking by methylation/expression relationships (ELMER) analysis***

In this analysis, we used profiles from 24 WS patients and 25 controls with both methylation and expression data sets. Using the ELMER R package (version 2.6.1) in supervised mode, the methylation and expression data were integrated to investigate GRNs and identify related master regulator transcription factors [7,8]. In short, for this analysis the processed methylation data β values, as described above, were used, combined with processed microarray expression data (GSE89594). Only distal probes were selected, defined as probes at least ± 2kb away from a transcription start site (TSS). The methylation and expression data of samples in both datasets (24 patients and 25 controls) were then combined into a MultiAssayExperiment [9], together with the group information, using hg19 coordinates and annotation, and linearizing the relationship between methylation and expression (through a log2(expression + 1) transformation). The outcome of the Limma analysis was used to determine differentially hypermethylated and hypomethylated distal probes, using the same criteria, but with recalculated FDR values based on the subset of distal probes. For these altered probes, the 20 closest genes (10 upstream and 10 downstream) were then identified and the association between probe methylation and gene expression was tested with a non-parametric Mann-Whitney U test, using the FDR for multiple testing correction. Significant probe–gene pairs were defined as pairs with an FDR < 0.01. The sets of genes associated with hyper- and hypomethylated probes were further investigated through a GO analysis with Enrichr [10]. Next, a transcription factor binding motif enrichment analysis was performed on the regions (± 250 bp) around the probes of significant hyper- and hypomethylated probe–gene pairs using a Fisher’s exact test. A motif was considered enriched if the odds ratio was > 1.1, it occurred > 10 times in the probe set regions, and the FDR was < 0.05. Finally, potential upstream master regulator transcription factors were determined by comparing the average methylation value of all probes associated with a motif with the expression values of genes annotated as a transcription factor with a Mann-Whitney U test. For this test it was assumed that a lower transcription factor expression corresponds to increased methylation levels [8]. After ranking by p-value, the top 5% of transcription factors were considered master regulators.

***1.3 meQTL analysis***

Methylation quantitative trait loci were analyzed based on Hannon et al. [11].

***1.4 Comparative genomic analysis***

Comparative genomic analysis was performed in Ensembl Genome Browser release 95. Gene orthology predictions were generated using the ensemble gene tree method.

**
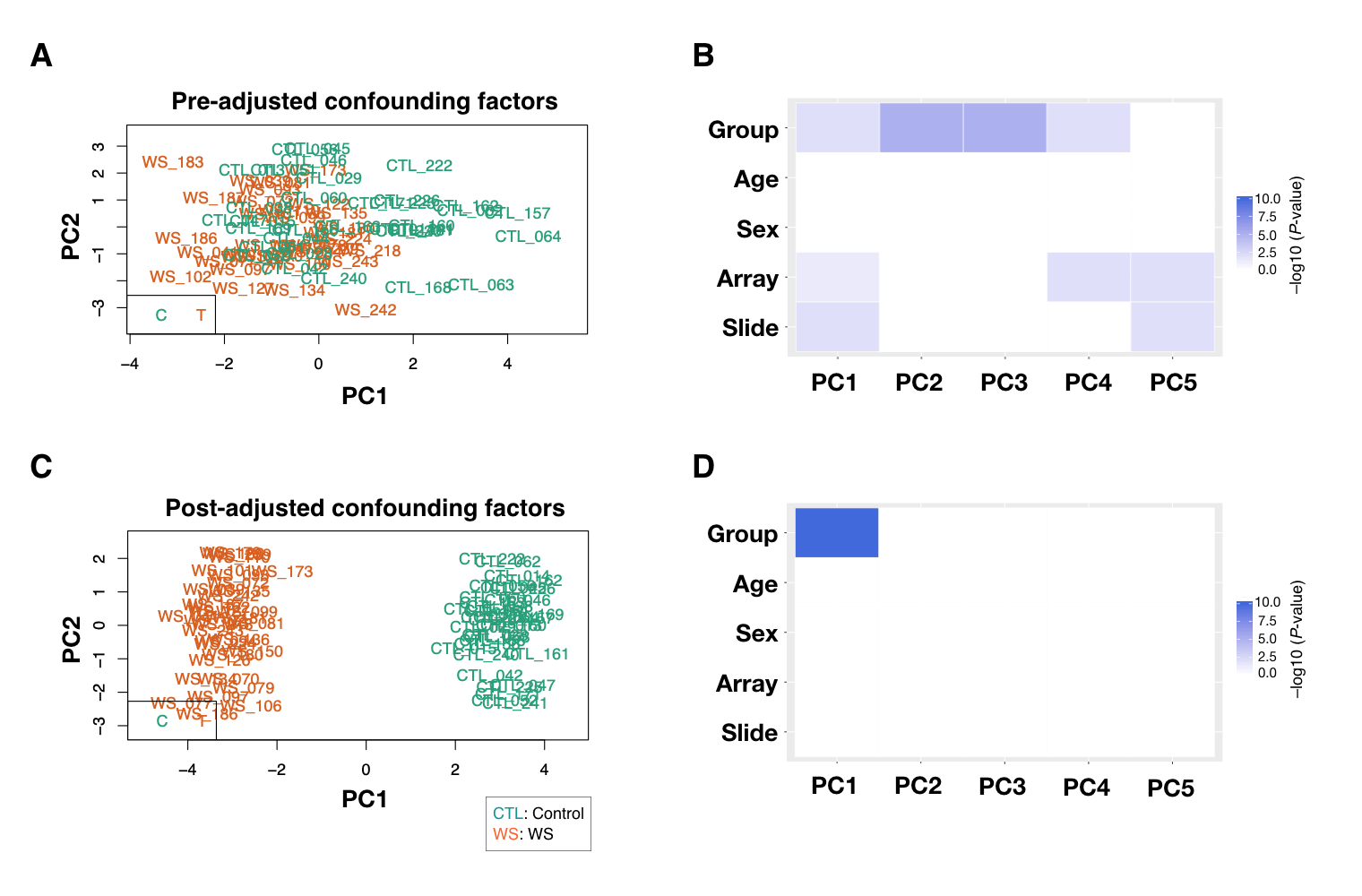
2. Supplementary Figures**

**Supplementary Figure 1.** **Assessment of confounding factors**. Before **(A and B)** and after **(C and D)** adjustment for confounding factors. (**A and C**) Multidimensional scaling plots visualized the similarity of samples based on the top 1000 most variable probes amongst all samples. (**B and D**) The singular value decomposition method to assess the potential confounding factors including age, sex, array positions, and slide.

.

**
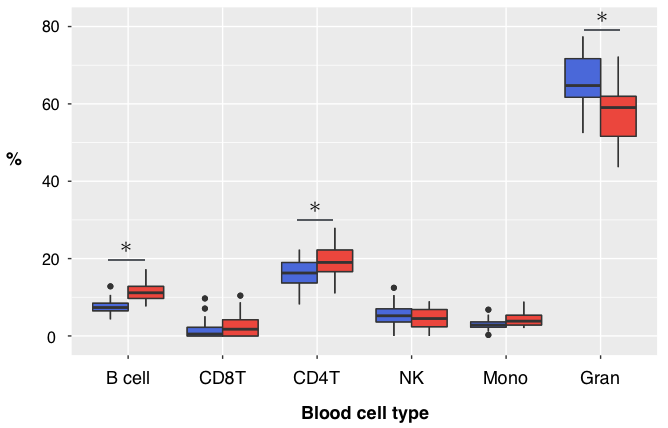
**

**Supplementary Figure 2.** **Blood cell-type composition of WS patients and controls**. The proportion of B cells, CD8^+^ T cells, CD4^+^ T cells, natural killer (NK) cells, monocytes (Mono), and granulocytes (Gran) were estimated based on DNA methylation profiles. *, Bonferroni corrected p-values were considered statistically significant. WS patients (red) and controls (blue) are shown as box plots. The error bars represent the standard deviation.

**
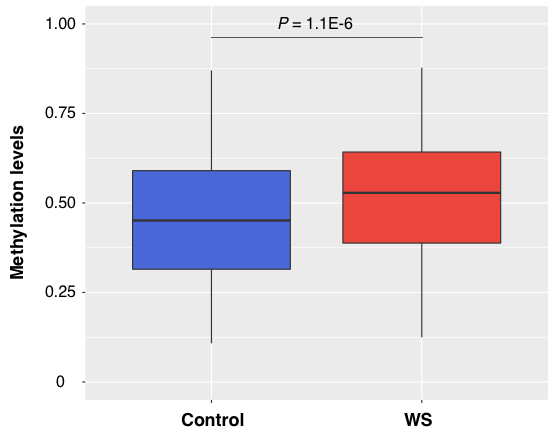
**

**Supplementary Figure 3. Global differences in the methylation levels of DMPs between WS patients and controls.** DMPs with an FDR < 0.05 and a |Δβ value| > 0.1. P-values were determined by Mann-Whitney U test.


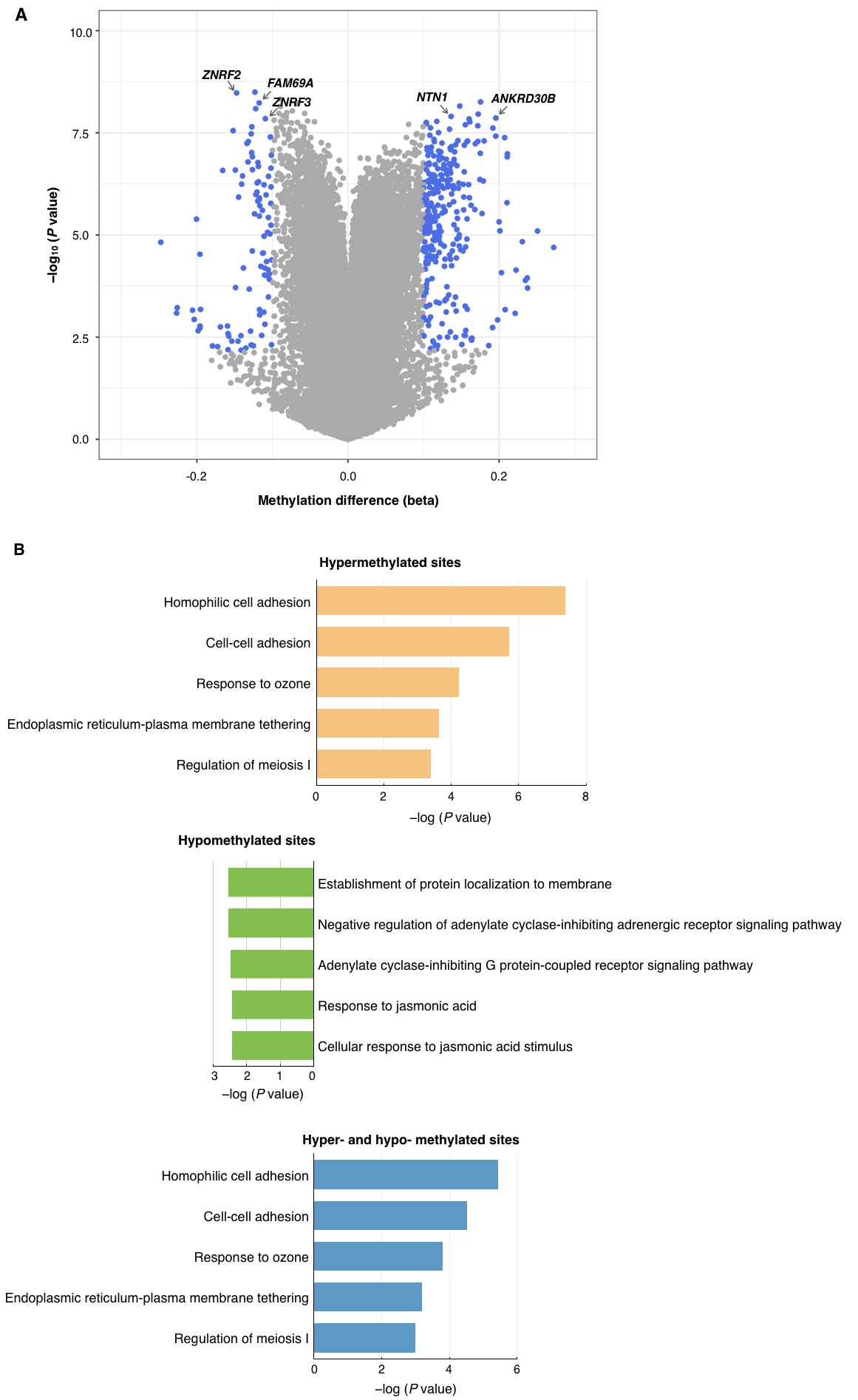


**Supplementary Figure 4. Differential methylation between WS patients and controls.** (**A**) Volcano plot of CpG sites based on methylation differences and p-values. Blue dots indicate DMPs with an FDR < 0.05 and a |Δβ value| > 0.1. (**B**) GO of top five biological process terms enriched in hyper-, hypo-, and both methylated positions.

**
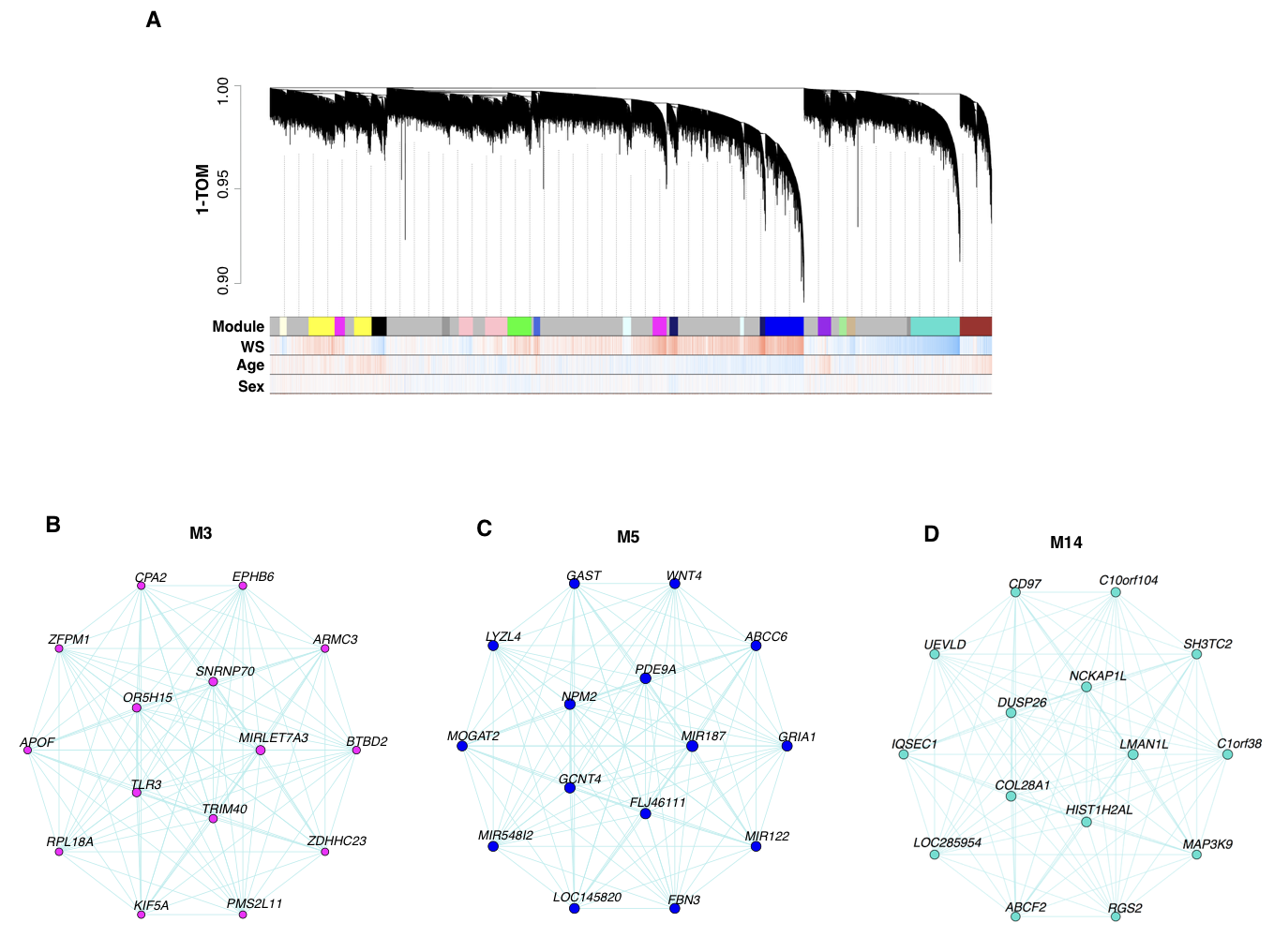
**

**Supplementary Figure 5. Co-methylation modules associated with WS, as identified by WGCNA.** (**A**) A dendrogram showing 16 distinct co-methylation modules based on a dissimilarity measure (1-TOM). (**B, C, and D**) Co-methylation networks among hub genes in module: (**B**) M3, (**C**) M5, and (**D**) M14.

**
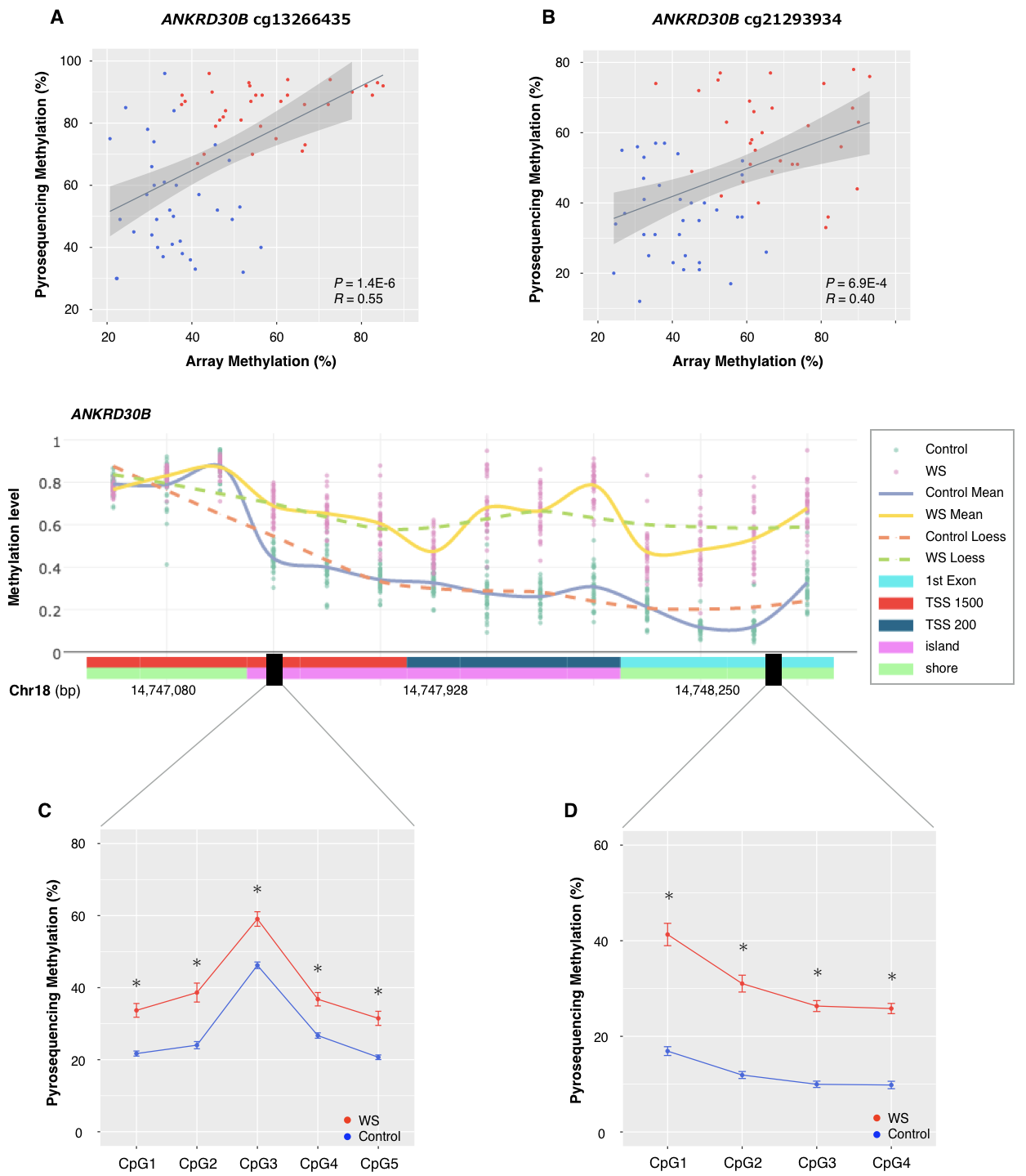
**

**Supplementary Figure 6. Pyrosequencing validation of CpG sites in *ANKRD30B*.** (**A and B**) Correlation of the methylation status of two different CpG sites in *ANKRD30B*: (**A**) cg13266435 and (**B**) cg21293934 between the methylation array and the pyrosequencing assays. Spearman’s correlation coefficient (R) and p-values are indicated. (**C and D**) Validation of CpG sites in *ANKRD30B*. A predesigned *ANKRD30B* PyroMark CpG assay was selected from the Qiagen GeneGlobe Web Portal (Cat# PM00071449 and PM00071456); (**C**) Five sites of 200–1500 bases upstream from the transcriptional start site (TSS 1500) were assayed by PM00071449. (**D**) Four sites in exon 1 were assayed by PM00071456. Red indicates WS patients while blue indicates controls. *p < 0.05 by Mann-Whitney U test.


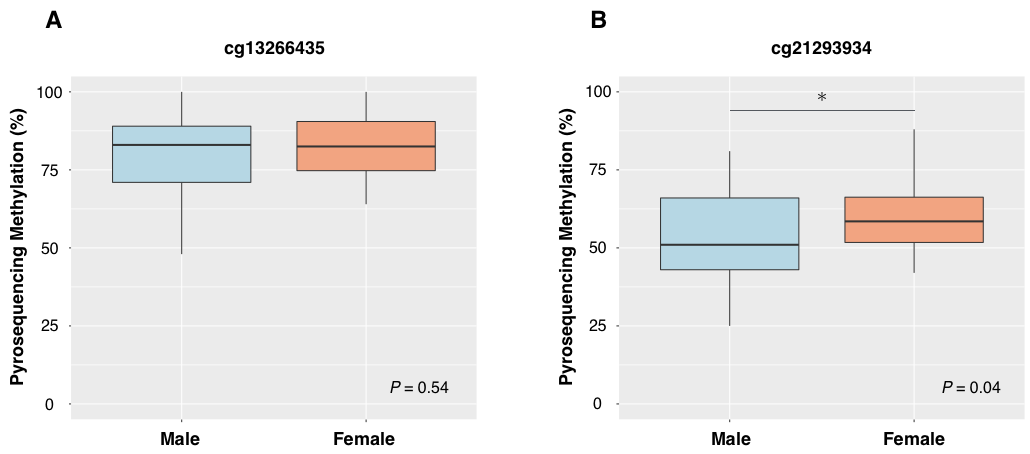


**Supplementary Figure 7. Effects of sex on DNA methylation in *ANKRD30B*.** Box plots represent methylation levels of the CpG sites in *ANKRD30B* (**A**) cg13266435 and (**B**) cg21293934. as measured by pyrosequencing using the complete WS set including male (n = 41) and female patients (n = 49). Age distribution between males and females was not significantly different. P-values were determined by Mann-Whitney U test.


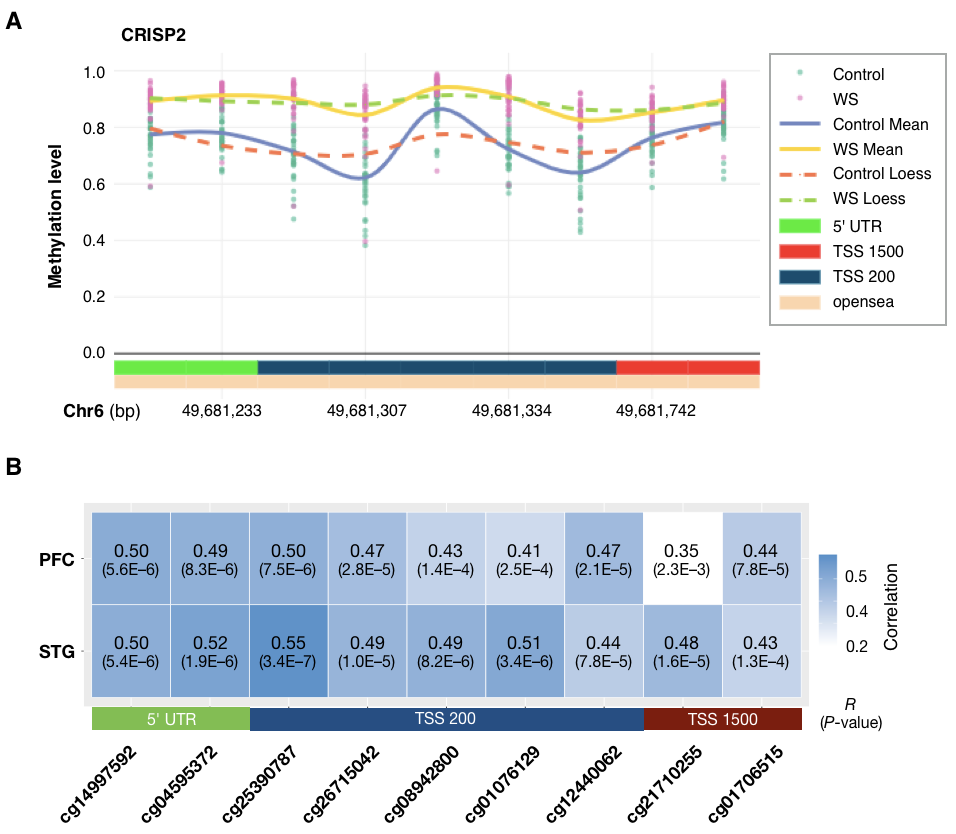


**Supplementary Figure 8. DNA methylation in *CRISP2*.** (**A**) Scatter plots with loess lines showing *CRISP2* methylation in WS patients and control individuals. Data points depict the methylation levels of each probe in individual samples based on the methylation array data. (**B**) Correlation of the methylation status of CpG sites in *CRISP2* between blood samples and the PFC and STG tissues in the brain. Pearson’s correlation coefficient (R) and p-values are indicated. 5' UTR, 5' untranslated region; TSS1500, 200–1500 bases upstream from the transcriptional start site; TSS200, 0–200 bases upstream from the transcriptional start site; opensea, far away from CpG islands.

**3. Supplemental References**

1. Kimura R, Swarup V, Tomiwa K, et al. Integrative network analysis reveals biological pathways associated with Williams syndrome. J Child Psychol Psychiatry. 2019;60(5):585-98.

2. Parras A, Anta H, Santos-Galindo M, et al. Autism-like phenotype and risk gene mRNA deadenylation by CPEB4 mis-splicing. Nature*.* 2018;560(7719):441.

3. Demontis D, Walters RK, Martin J, et al. Discovery of the first genome-wide significant risk loci for attention deficit/hyperactivity disorder. Nat Genet*.* 2019;51(1):63.

4. Savage JE, Jansen PR, Stringer S, et al. Genome-wide association meta-analysis in 269,867 individuals identifies new genetic and functional links to intelligence. Nat Genet*.* 2018;50(7):912.

5. Nardone S, Sams DS, Zito A, Reuveni E, Elliott E. Dysregulation of cortical neuron DNA methylation profile in autism spectrum disorder. Cereb Cortex*.* 2017;27(12):5739-5754.

6. Strong E, Butcher DT, Singhania R, et al. Symmetrical Dose-Dependent DNA-Methylation Profiles in Children with Deletion or Duplication of 7q11.23. Am J Hum Genet*.* 2015;97(2):216-227.

7. Hazelett DJ, Gull N, Coetzee SG, et al. ELMER v.2: an R/Bioconductor package to reconstruct gene regulatory networks from DNA methylation and transcriptome profiles. Bioinformatics.2019;35(11):1974-1977.

8. Yao L, Shen H, Laird PW, Farnham PJ, Berman BP. Inferring regulatory element landscapes and transcription factor networks from cancer methylomes. Genome Biol*.* 2015;16(1):105.

9. Ramos M, Schiffer L, Re A, et al. Software for the integration of multiomics experiments in Bioconductor. Cancer Res*.* 2017;77(21):e39-e42.

10. Kuleshov MV, Jones MR, Rouillard AD, et al. Enrichr: a comprehensive gene set enrichment analysis web server 2016 update. Nucleic Acids Res*.* 2016;44(W1):W90-W97.

11. Hannon E, Lunnon K, Schalkwyk L, Mill J. Interindividual methylomic variation across blood, cortex, and cerebellum: implications for epigenetic studies of neurological and neuropsychiatric phenotypes. Epigenetics*.* 2015;10(11):1024-1032.
